# Supplementary material for: Causal Relationship between Aging and Anorexia Nervosa: A White-Matter-Microstructure-Mediated Mendelian Randomization Analysis
Source: Biomedicines. 2024 Aug 16;12(8):1874. doi: 10.3390/biomedicines12081874 (PMC11351342; doi:10.3390/biomedicines12081874)
Supplement: Supplementary file 1 [file biomedicines-12-01874-s001.zip › Supplementary Figure/Figure S4/Singe SNP plot of MR analysis for TL to ubm-b-2007.pdf]

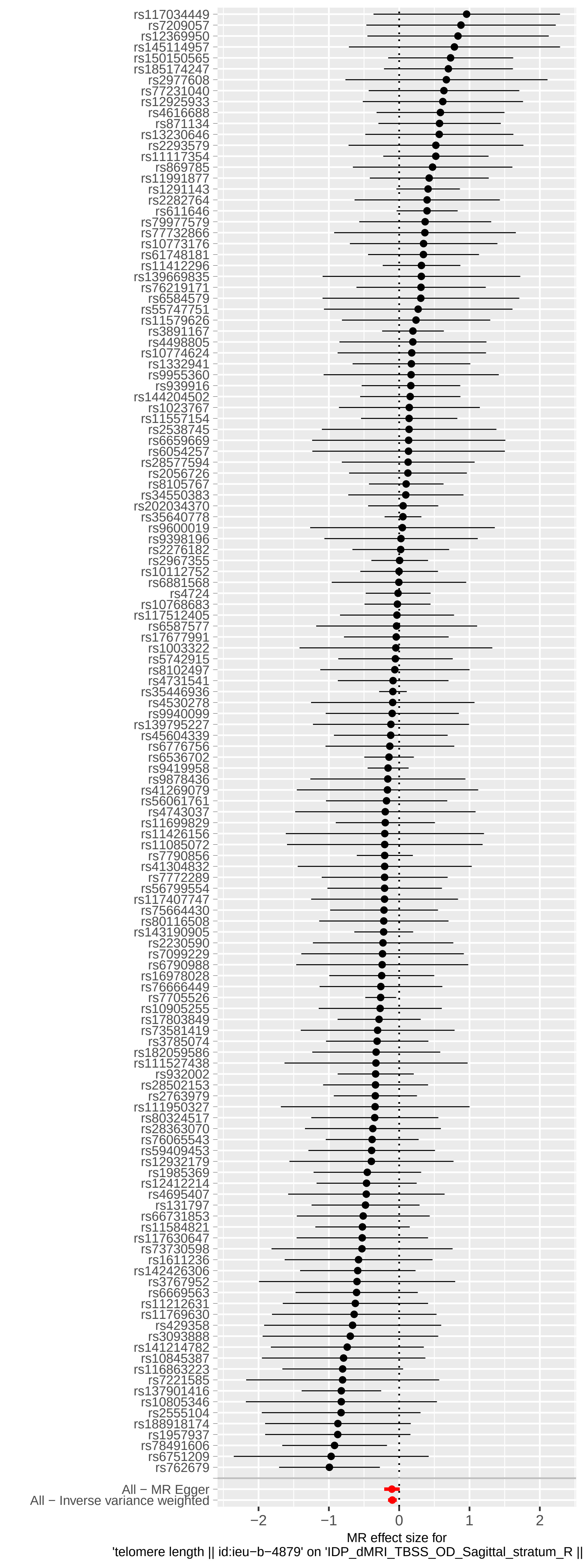
rs117034449
rs7209057
rs12369950
rs145114957
rs150150565
rs185174247
rs2977608
rs77231040
rs12925933
rs4616688
rs871134
rs13230646
rs2293579
rs11117354
rs869785
rs11991877
rs1291143
rs2282764
rs611646
rs79977579
rs77732866
rs10773176
rs61748181
rs11412296
rs139669835
rs76219171
rs6584579
rs55747751
rs11579626
rs3891167
rs4498805
rs10774624
rs1332941
rs9955360
rs939916
rs144204502
rs1023767
rs11557154
rs2538745
rs6659669
rs6054257
rs28577594
rs2056726
rs8105767
rs34550383
rs202034370
rs35640778
rs9600019
rs9398196
rs2276182
rs2967355
rs10112752
rs6881568
rs4724
rs10768683
rs117512405
rs6587577
rs17677991
rs1003322
rs5742915
rs8102497
rs4731541
rs35446936
rs4530278
rs9940099
rs139795227
rs45604339
rs6776756
rs6536702
rs9419958
rs9878436
rs41269079
rs56061761
rs4743037
rs11699829
rs11426156
rs11085072
rs7790856
rs41304832
rs7772289
rs56799554
rs117407747
rs75664430
rs80116508
rs143190905
rs2230590
rs7099229
rs6790988
rs16978028
rs76666449
rs7705526
rs10905255
rs17803849
rs73581419
rs3785074
rs182059586
rs111527438
rs932002
rs28502153
rs2763979
rs111950327
rs80324517
rs28363070
rs76065543
rs59409453
rs12932179
rs1985369
rs12412214
rs4695407
rs131797
rs66731853
rs11584821
rs117630647
rs73730598
rs1611236
rs142426306
rs3767952
rs6669563
rs11212631
rs11769630
rs429358
rs3093888
rs141214782
rs10845387
rs116863223
rs7221585
rs137901416
rs10805346
rs2555104
rs188918174
rs1957937
rs78491606
rs6751209
rs762679
All − MR Egger
All − Inverse variance weighted
−2
−1
0
1
2
MR effect size for
'telomere length || id:ieu−b−4879' on 'IDP_dMRI_TBSS_OD_Sagittal_stratum_R ||
